# Cardiac Arrest Mortality and Disposition Patterns in United States Emergency Departments

**DOI:** 10.3390/jcm13185585

**Published:** 2024-09-20

**Authors:** Kenneth M. Zabel, Mohammed A. Quazi, Katarina Leyba, Alexandra C. Millhuff, Mikel Madi, Wilfredo Henriquez Madrid, Aman Goyal, Muhammad Ibraiz Bilal, Amir H. Sohail, Shazib Sagheer, Abu Baker Sheikh

**Affiliations:** 1 Department of Internal Medicine, University of New Mexico, Albuquerque, NM 87131, USA; kmzabel@salud.unm.edu (K.M.Z.); amillhuff@salud.unm.edu (A.C.M.); 2 Department of Biostatistics and Mathematics, University of New Mexico, Albuquerque, NM 87113, USA; maquazi@salud.unm.edu; 3 Department of Internal Medicine, University of Colorado, Aurora, CO 80045, USA; katarinaleyba@gmail.com; 4 Department of Medicine, American University of Beirut, Beirut 1107-2020, Lebanon; 5 Division of Cardiology, University of New Mexico, Albuquerque, NM 87106, USA; whenriquez@salud.unm.edu; 6 Department of Internal Medicine, Seth GS Medical College and KEM Hospital, Mumbai 400012, India; amanmgy@gmail.com; 7 Department of Internal Medicine, Allegheny Health Network, Pittsburgh, PA 15212, USA; ibraiz47@hotmail.com; 8 Department of Surgical Oncology, University of New Mexico, Albuquerque, NM 87131, USA; asohail@salud.unm.edu; 9 Division of Interventional Cardiology, Newark Beth Israel Medical Center, Newark, NJ 07112, USA; shazibcheema@gmail.com

**Keywords:** cardiac arrest, emergency department, mortality, disposition

## Abstract

**Background**: Despite resuscitative efforts, cardiac arrest (CA) continues to result in high mortality and poor prognosis. However, a gap remains in understanding the comparative outcomes of efforts in emergency departments (ED) over recent years. This study evaluated patients with CA during ED visits, with a particular focus on outcomes of mortality and transition of care. **Methods**: We conducted a retrospective cohort analysis using the National Emergency Department Sample (NEDS) database. The study population included patients aged 18 years or older who visited the ED between January 2016 and December 2020. Statistical analysis of patients and hospital characteristics included chi-squared tests for independence and multivariable logistic regression models to report the associations of factors with mortality in the ED and disposition from the ED. The primary outcome measured was mortality in the ED, and the secondary outcome included transition of care. **Results**: A total of 699,822,424 ED visits occurred between 2016 and 2020, with 1,414,060 (0.20%) CAs. The survival rate from CA ranged from 24.6% to 28.1%. In 2020, the rate of ED CA increased to 0.27%, with an inpatient mortality rate of 58.8%. There was no significant difference in mortality between sexes (*p* = 0.690). There was a trend for higher mortality in the ED among patients who were self-paid. Notably, the odds of transfer from the ED to other hospitals were significantly lower in minority groups. **Conclusions**: Our results showed significant disparities in ED mortality and patient disposition following cardiac arrest, highlighting the need for equitable healthcare resources and policies.

## 1. Introduction

Cardiac arrest (CA) and sudden cardiac death (SCD) are associated with high mortality and poor prognosis, despite the myriad medical advances in recent decades. CAs are classified into out-of-hospital CA (OHCA) and in-hospital CA (IHCA). OHCA incidence increased from 47 per 100,000 in 2008 to 66 per 100,000 in 2015 [1]. Similarly, the rates of IHCA have remained high, with recent analyses finding rates of 9–10 IHCAs per 1000 hospitalizations in the United States [2]. Moreover, effective measures for CA remain limited, and the only strategies with a proven mortality benefit are early, high-quality chest compressions and early defibrillation in the appropriate setting [3,4]. Even though these interventions have improved survival rates, the overall survival in CA remains low, with the survival rate of CA increasing from just 5.7% in 2005 to 8.3% in 2012 [5].

While recent studies have used the National Emergency Department Sample (NEDS) database to evaluate CA, they have primarily investigated etiologies and predictors of CA in the ED [6]. In this analysis, we utilize the NEDS database to analyze the most recent data on patients who suffered CA or required cardiopulmonary resuscitation (CPR) during their ED visit between 2016 and 2020, with a particular focus on mortality and disposition from the ED.

## 2. Materials and Methods

This retrospective observational cohort study used data obtained from the NEDS database. Patient and hospital characteristics were recorded, and the patients were tracked through their emergency room visits using the International Classification of Diseases Version 10 (ICD-10). 

### 2.1. Study Population Inclusion and Exclusion Criteria

From January 2016 to December 2020, this study included patients aged ≥18 years who visited the ED and experienced CA or required CPR during their ED visit based on ICD-10 codes (Supplementary Table S1). Exclusion criteria included patients under 18 years of age, those with incomplete data, those with a do-not-resuscitate (DNR) code status, and individuals who experienced CA or required CPR outside of the ED.

### 2.2. Study Definitions and Variables

The NEDS database is the largest all-payer ED database in the United States [7]. Data collection began in 2006, allowing for the comparison of ED visits over time. Unweighted, the NEDS contains data from approximately 20–30 million ED visits for each year of the study period. Weighted, it estimates approximately 100–125 million ED visits for each year from 2016 to 2020.

Both patient and hospital characteristics were reported in the NEDS database and utilized in this study. Patient demographics, which were recorded based on the NEDS protocol, included age, sex, race, household income, insurance status, and comorbid medical conditions. Patients who experienced cardiac arrest were identified using the ICD-10 codes I462, I468, and I469. The ICD codes for disease, comorbidities, and complications are depicted in Supplementary Table S1.

Patients were categorized into three groups based on their disposition from the ED: admitted to the hospital as an inpatient, died in the ED, and discharged alive. The patient’s mortality status was also recorded as mortality in the ED or mortality after inpatient admission to the hospital. 

### 2.3. Outcomes

The primary outcome was mortality in the ED, and the secondary outcome was patient disposition from the ED.

### 2.4. Statistical Method

We built a multivariate logistic regression model in Python using patient (age, race, gender, insurance status, income and elixhauser comorbidies index)and hospital-related variables (teaching status, bed size, rural/urban and geographical region as predictors, to report the adjusted odds ratio (aOR) for mortality in the ED. The 95% confidence intervals (CI) were also reported, along with the *p*-values for statistical significance. The categorical variables were analyzed using the chi-square test for independence against the two cohort categories: in-hospital survival and in-hospital mortality. The *p*-values for the chi-square tests are also reported in the same table. The same tests for independence were repeated for the disposition categories in the ED (admitted inpatient, mortality in ED, discharged from ED and transfer from ED to other facility). Furthermore, we built another multivariable logistic regression model to analyze the association of the same patient- and hospital-related predictors from the previous model with transfer from the ED to other facility. The aOR for transfer from the ED is reported along with the 95% CI. 

## 3. Results

A total of 699,822,424 ED visits occurred between 2016 and 2020. The average annual ED visits between 2016 and 2019 were 144,136,064 (SD 692,822) per year. The number of ED visits decreased to 123,278,165 in 2020. During the study period, a total of 1,414,060 (0.20%) CAs occurred in the ED. The mean annual number of CAs in the ED was 270,539 (SD 6854) for the years 2016–2019. In 2020, this number increased to 331,906 (Table 1).

### 3.1. Demographics

A total of 1,414,060 patients were treated for CA in a hospital-based ED between 2016 and 2020. Sixty-one percent (*n* = 861,046) were male. The mean age was 66 years (SD 16.89) for females and 63 years (SD 16.79) for males. The majority of patients, (41.4%, *n* = 586,731) were 70 years of age or older, followed by those aged between 50 and 69 years (39.9% or 563,678).

Self-reported race and ethnicity data were available only for 2019–2020. Among the 42% of patients for whom race and ethnicity data were available, the largest group was white (*n* = 367,009; 25.42%), followed by Black (*n* = 127,230; 8.9%), Hispanic (*n* = 66,544; 4.8%), Other (*n* = 23,783; 1.7%), Asian and Pacific Islanders (*n* = 15,834; 1.1%), and Native American (*n* = 2521; 0.2%).

The majority of patients (*n* = 499,190; 35.3%) had a mean annual income of <USD 49,999, followed by patients with an annual income of USD 50,000–64,999 (26.0%; *n* = 369,425), and patients with an annual income of USD 65,000–85,999 (20.8%; *n* = 294,982). A total of 250,465 patients (17.9%) had an annual income of USD 86,000 or greater.

In terms of insurance, the largest group was Medicare (*n* = 777,920; 55%), while 12.8% (*n* = 178,950) had Medicaid coverage. A total of 18.9% (*n* = 267,487) had private insurance, and 10.4% (*n* = 144,059) were self-payers.

Baseline demographic characteristics of included patients are further outlined in Table 2.

### 3.2. Hospital Characteristics

Of the hospitals included in the study, the majority 45.2% (*n* = 630,898) were treated in hospitals in the South Atlantic region, followed by the Midwest (20.1%; *n* = 299,039), West (17.8%; *n* = 230,581), and Northeast region (16.9%; *n* = 253,543).

Sixty percent (*n* = 871,843) of the patients received care at metropolitan teaching hospitals, 26.7% (*n* = 337,624) at metropolitan non-teaching hospitals, and 13.2% (*n* = 204,595) at non-metropolitan hospitals (Table 2).

### 3.3. Primary Outcome—ED Mortality

Of those who suffered a CA, 52.6% died in the ED. The combined ED and inpatient mortality was 71.9–73.8% per year between 2016–2019 and increased to 75.4% in 2020, concurrent with the higher number of CAs treated in the ED in 2020. This can be accounted for by an increase in inpatient mortality, which rose from 49.2–52.9% in 2016–2019 to 58.8% in 2020. 

There was no significant difference in mortality between men and women (aOR 1, Cl 0.97–1.02, *p* = 0.690. Risk of ED mortality increased with increasing age. Relative to patients aged 18–29, the 30–49-year group had a significantly higher risk of mortality (aOR 1.3, CI 1.22–1.39, *p* < 0.001), and the risk was more pronounced with advanced age (50–69 years: aOR 1.71, CI 1.60–1.82, *p* < 0.001; >70 years: aOR 2.38, CI 2.23–2.55, *p* < 0.001).

Compared to white patients, Asian or Pacific Islander (OR 0.79, CI 0.73–0.85, *p* < 0.001), Black (OR 0.9, CI 0.92–0.99, *p* = 0.008), and Hispanic (OR 0.81, CI 0.77–0.84, *p* < 0.001) patients had lower mortality risk in the ED.

Mortality rates also varied according to income and payer status, with higher income being associated with higher mortality in the ED. Relative to patients with a median annual household income <USD 49,000 annually, the USD 65,000–85,999 (OR 1.09, CI 1.05–1.13, *p* < 0.001) or >USD 86,000 (OR 1.12, CI 1.07–1.16, *p* < 0.001) groups had higher odds of mortality in ED. Patients who were self-payers (OR 1.77), no charge, or who had Medicare had significantly higher ED mortality rates compared to patients insured by Medicaid, while patients with private insurance were at comparatively lower risk of dying (OR 0.95, CI 0.91–1.0, *p* < 0.001).

Relative to patients treated in the Midwest, patients treated at hospitals in the West geographic region had lower rates of mortality (OR: 0.82, 95% CI: 0.79–0.86, *p* < 0.001), while the Northeast and South Atlantic regions did not have a significantly different mortality rate.

There was a significant relationship between Elixhauser comorbidity score and ED mortality. Patients with greater than four Elixhauser comorbidities were more likely to die in the ED than patients with four or fewer Elixhauser comorbidities (OR 1.18 95% CI 1.01–1.137, *p* = 0.035).

The predictive variables of ED mortality are further detailed in Table 3. The inpatient mortality rates are provided in Supplementary Table S1.

### 3.4. Common Primary Etiologies for Cardiac Arrest in ED

Cardiac arrest was the most common primary diagnosis in the emergency department, comprising 54% (*n* = 761,889) of cases, with a high mortality rate of 86.5% (*n* = 659,089). Respiratory failure accounted for 3.7% (*n* = 52,223) of cases with a mortality rate of 28.8% (*n* = 15,088). Other conditions, such as gastrointestinal bleed (0.3%, *n* = 4240), syncope (0.2%, *n* = 2905), and ST-elevation myocardial infarction (STEMI) (3.3%, *n* = 46,248), had lower mortality rates of 7.2%, 5.13%, and 3.83%, respectively. COVID-19 represented 1.2% (*n* = 16,952) of cases with a 3.69% mortality rate. Ventricular fibrillation (1.6%, *n* = 22,046) and non-ST-elevation myocardial infarction (NSTEMI) (1.7%, *n* = 24,373) had mortality rates of 3.57% and 1%, respectively. Sepsis, despite accounting for 8.9% (*n* = 125,303) of cases, had a relatively low mortality rate of 0.79% (*n* = 990). These results illustrate varying mortality rates across different primary diagnoses in the ED Table 4.

### 3.5. Secondary Outcome—Disposition from the ED

**Figure 1:**
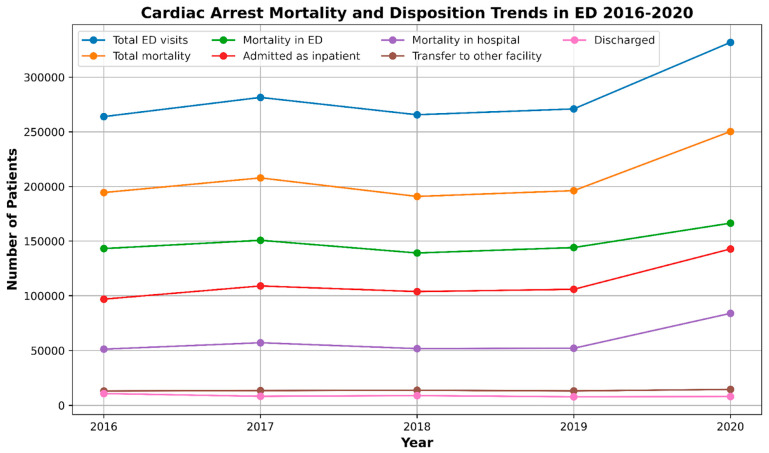
Trends in mortality due to cardiac arrest and disposition in emergency departments (EDs) from 2016 to 2020.

Of the 1,414,060 CA ED visits for patients in the study period, 52.6% (*n* = 743,642) died in the ED. Forty-seven percent survived. Among these, 39.5% (*n* = 558,364) were admitted to the hospital, 4.8% (*n* = 68,182) were transferred to another facility/hospital, and 3.1% (*n* = 43,872) were discharged. The inpatient mortality was 53% (*n* = 296,119) (Figure 1) Table 1. 

A statistically significant difference (*p* < 0.001) was noted regarding disposition to different destinations across age groups. The majority of patients admitted to the inpatient unit were aged between 50 and 69 years (42.8%) and 42% were female. Meanwhile, patients in the 18–29 age group were the most commonly discharged (4.6%). Compared to other age groups, younger <30 years and elderly patients aged above 70 were more likely to die in the ED, with mortality rates of 56.4% and 55.5%, respectively (*p* < 0.001). Across different insurance types, the prevailing majority of patients were insured through Medicare irrespective of their disposition, except for those who died in the ED, who were primarily self-payers (70.5%, *p* < 0.001). Of patients with >4 Elixhauser comorbidities, 92% had inpatient admission; see Table 5. 

Regarding the hospital region, the majority of patients transferred to other hospitals were treated in Midwest hospitals (6.1%) whereas hospitals in the Northwest region predominantly had inpatient admissions (45.6%, *p* < 0.001). Most of the patients transferred to other facilities were from non-metropolitan or micropolitan and micropolitan areas (19.6% and 15.3% *p* < 0.001) Table 5.

In regards to transfer vs. inpatient admission odds ratios from the EDs, compared to men, women were more likely to be transferred versus admitted (OR 1.08, CI 1.01–1.15, *p* = 0.029). Patients aged between 50 and 69 years were more likely to be admitted as inpatients compared to younger patients aged between 18 and 29 years (OR 1.36, CI 1.17–159 *p* < 0.001). Odds of transfer were significantly lower for patients who identified as Asian or Pacific Islander, Black, or Hispanic (OR 0.47, 0.80, and 0.64, respectively, with *p* < 0.001 for all comparisons). Relative to patients with a median household income of <USD 49,999, patients with median household incomes in the range of USD 50,000–64,999 or USD 65,000–85,999 were more likely to be transferred to another facility, with patients in the USD 50,000–64,999 range having an OR of 1.30 (CI 1.20–1.41, *p* < 0.001) and patients in the USD 65,000–85,999 range having an OR of 1.17 (CI 1.06–1.29, *p* = 0.002).

Rates of inpatient admission also varied significantly by hospital urban–rural designation, with patients treated in micropolitan areas, non-urban residual, and small metropolitan areas all more likely to be transferred as compared to large metropolitan areas (OR 2.1, 6.8, and 1.2, respectively, *p* < 0.001). Based on the hospital status, patients were more likely to be transferred if they were treated at metropolitan non-teaching or urban hospitals compared to those treated at metropolitan teaching hospitals (OR 4.94 and 8.78, respectively, *p* < 0.001).

Patients with a higher comorbidity burden were more likely to be transferred than admitted, with patients with more than four Elixhauser comorbidities having an increased rate of transfer relative to patients with four or fewer comorbidities (OR 2.59, CI 1.77–3.80, *p* < 0.001).

The aOR for transfer from EDs against inpatient admission is further detailed in Table 6.

## 4. Discussion

This study represents the next iteration in the continuous evaluation of CA from the NEDS database. When compared to previous studies, the mortality rate reported in this study matched previous rates, with dismal survival rates across all age groups. Type of insurance had an impact on mortality, as patients who were self-pay had significantly higher rates of mortality, which was also reflected in prior studies [8,9]. From 2016 to 2018, the aggregated survival rate from cardiac arrest was 24.7% [9]. Our study found that between the years of 2016 and 2020, the survival rate from cardiac arrest ranged from 24.6–28.1%. 

It is important to acknowledge the potential impact of the COVID-19 pandemic on ED mortality rates during this time period. While the number of annual ED visits was fairly constant from 2016 to 2019, this number decreased by over 20 million in the year 2020. This is consistent with the results of prior studies utilizing different datasets to compare rates of ED visits during the COVID-19 pandemic [10,11]. One can postulate that this decrease is due to concerns about contracting COVID-19 and the impetus to quarantine. Interestingly, an analysis by Daoud and Ronen found that this decrease held true when examining urgent or life-threatening causes for ED visits as well as non-urgent causes. This offers one potential explanation for the increase in the rate of CA in the ED in 2020. Whereas the rate of CA among all patients treated in the ED was consistently 0.18–0.19% between 2016 and 2019, the rate of ED CA rose to 0.27% in 2020. In the context of patients deferring medical care for both non-urgent and urgent causes, it is possible that patients delayed seeking care until they were in a more critical condition, ultimately leading to a higher rate of CA in 2020. Concordant with this, the rate of inpatient admission to the same hospital and the rate of ED mortality for all patients treated in the ED also increased in 2020 (to 0.12% from 0.07–0.08% in the preceding four years and 0.14% from 0.10% in the preceding four years, respectively), suggesting patients were presenting at a more advanced stage of illness. Additionally, the inpatient mortality for CA patients rose from 49.2–52.9% during 2016–2019 to 58.8% in 2020, further supporting the hypothesis that the pandemic led to delayed care and more severe presentations of illness.

Regarding mortality, it should be noted that this study evaluated only immediate mortality rates in the ED in detail and not a patient’s overall hospital course. The higher mortality rate in the older population is expected and consistent with the prior study [9]. This study found that patients who identify as Asian American, Black, or Hispanic had lower ED mortality compared to white patients. However, accuracy in self-reporting racial subgrouping has historically been poor due to inadequate choices on intake paperwork and inherent biases present in clinical and non-clinical staff [12]. These forms of systematic racial prejudice have not been satisfactorily addressed [13,14]. Moreover, there is a possibility that minority racial groups are more likely to die from out-of-hospital cardiac arrest and thus are not represented in the ED patient population. In our analysis, race was unreported for over half (58%) of the study population, as self-reported race data were only available for years 2019–2020 and not for 2016–2018. Thus, this correlation should be interpreted with caution. Prior studies show that post-IHCA, Black and Hispanic patients have lower rates of neurologic recovery compared to white patients [15,16]. Variability in patient care during ED and inpatient settings for CA and post-CA may explain why these racial differences exist, though further study is needed to better understand this disparity and identify opportunities to improve outcomes for all patients.

Notably, our study does not index whether CA experienced by the patient is a primary or secondary diagnosis. Patients with a secondary diagnosis of CA have significantly higher survival compared to a primary diagnosis of CA (41.9 vs. 15.7%) [17]. The effect of this possible confounder is not analyzed in our study, and future analyses may further classify CA as primary or secondary in nature. Other limitations in this study include its retrospective nature; in this way, causation cannot be inferred and this study is primarily hypothesis-driving. This study also relies heavily on reported coding (ICD/CPT), which does not always accurately capture the complete clinical picture for a patient’s case. There is also limited information related to patient clinical context, such as the etiology of cardiac arrest and cardiac rhythm upon the time of arrest. Therefore, a subgroup analysis based on these factors, which are known predictors of survival, was not feasible [18,19,20].

Furthermore, our study excluded patients with a DNR code status. If patients had specified these wishes prior to experiencing CA in the ED and were not resuscitated, mortality for these patients would be certain. Prior studies have noted lower rates of DNR or CMO code statuses for Black patients compared to white patients [21,22,23]. However, this finding would contribute to higher mortality rates in Black patients, which was not observed in our study but has been reported in prior studies of in-hospital CA outcomes [16].

This analysis found that risk of ED mortality after CA was greater among patients of higher median household income. Relative to patients with a median household income of <USD 49,000 annually, patients with a median household income of USD 65,000–85,999 and >USD 86,000 were at increased risk of death in the ED (OR 1.09 with *p* < 0.001 and OR 1.12 with *p* < 0.001, respectively). This finding diverges from the findings of prior studies, which have consistently found increasing income to be associated with improvements in mortality [24,25,26,27]. One possible explanation for this apparent discrepancy is that income tends to rise with age, and this analysis found that increasing age was significantly associated with increasing mortality risk [28]. Thus, the impact of median household income on mortality in this analysis may be confounded by the strong relationship between income and age. 

Our study found Asian or Pacific Islander, Black, and Hispanic patients had lower ED mortality and lower odds of transfer to another hospital compared to white patients. One plausible explanation for the lower odds of transfer for these racial groups could be differences in hospital resources and capabilities. Hospitals that predominantly serve minority communities may have fewer resources or lower capacity for transfers, leading to higher rates of inpatient admissions. Additionally, socioeconomic factors and systemic biases may influence clinical decision-making, with minority patients being less likely to be transferred due to perceived or actual barriers to accessing higher levels of care [28]. Cultural and language barriers might also play a role, as healthcare providers may be less inclined to transfer patients if they believe that continuity of care will be better maintained in the current facility [29,30]. Furthermore, minority patients might have lower rates of private insurance, which can limit their options for transfer to certain hospitals, thus influencing the decision to admit them as inpatients rather than transfer them to another facility.

When CA is identified as a primary diagnosis in the ED, it is accompanied by a notably high mortality rate of 86.5%. This finding is consistent with prior literature, which indicates that primary cardiac arrests are associated with worse outcomes due to the immediate and severe impact on cardiac function [2,5]. In contrast, other prevalent etiologies contributing to CA, such as respiratory failure (28.8%), gastrointestinal bleed (7.2%), STEMI (3.83%), and sepsis (0.79%), demonstrated varying mortality rates that likely reflect differences in disease acuity and potential reversibility in the ED context. Secondary cardiac arrests, where the underlying cause may be more amenable to early intervention (e.g., drug overdose, respiratory failure), generally show better outcomes due to the possibility of reversing the precipitating condition. These findings underscore the importance of developing targeted interventions and optimizing resource allocation based on the specific underlying etiology of CA to enhance patient outcomes. Moreover, recognizing the diversity of primary diagnoses provides a foundation for more nuanced risk stratification and tailored management strategies for patients presenting with CA in the ED. Future research should prioritize the creation of evidence-based protocols that address the distinct needs of these subgroups to further mitigate mortality risks. While this study provides robust population-level insights, the integration of more granular clinical data, such as initial cardiac rhythm and time to intervention, would be essential to elucidate the variability in outcomes and refine clinical care pathways.

Our study contributes valuable insights into the patterns and outcomes of cardiac arrest in the ED, particularly in the context of the COVID-19 pandemic. We observed significant variations in ED mortality based on insurance type, socioeconomic status, and racial/ethnic backgrounds, underscoring the need to address systemic disparities in healthcare. Additionally, our comprehensive analysis of patient disposition—examining age groups, race, gender, region, insurance status, and hospital size—provides a novel perspective not previously explored in depth. Our study highlighted the lower odds of transfer versus inpatient admission for minority groups, revealing critical differences in hospital resources and decision-making processes. Future research should explore these complex factors further to develop strategies that enhance patient outcomes across diverse populations.

### Limitations

This study has several limitations that should be acknowledged. The retrospective nature of the study, using the NEDS database, limits the ability to infer causation, as it relies on pre-existing data and coding accuracy, which may not fully capture the clinical nuances of each case. The NEDS database does not provide detailed information on clinical parameters such as the etiology of cardiac arrest, initial cardiac rhythm, or interventions performed, which are critical factors influencing outcomes. Additionally, the scope of this dataset is limited to EDs and inpatient mortality and does not include information on longer-term outcomes such as 30-day or 90-day survival rates. The racial and ethnic data were self-reported and only available for a subset of the study period (2019–2020), leading to potential misclassification and limiting the generalizability of findings related to racial disparities. The study also does not differentiate between primary and secondary diagnoses of cardiac arrest, which have different prognostic implications. While socioeconomic factors and insurance status were included, they may not fully account for all variables impacting patient outcomes. Lastly, the reliance on administrative data means that unmeasured confounders could influence the results, and the findings may not be generalizable to non-hospital-based emergency care settings. Future research should aim to include more comprehensive clinical data and explore the impacts of these limitations in more detail.

## 5. Conclusions

Our study leverages the NEDS database to provide a comprehensive analysis of ED mortality and patient disposition following cardiac arrest, revealing the significant impacts of socioeconomic and racial factors, as well as the exacerbating effects of the COVID-19 pandemic. Despite the limitations related to the retrospective nature and data constraints, our findings emphasize the urgent need for equitable healthcare resources and policies that address disparities in patient outcomes. Continued research and policymaking efforts are essential to improve survival rates, patient disposition, and overall care for all patients experiencing cardiac arrest.

## Figures and Tables

**Table 1:** Total emergency department (ED) visits, dispositions, and mortality rates in patients with cardiac arrest from 2016 to 2020.

Variables	2016	2017	2018	2019	2020	Total
Total ED visits	144,842,742	144,814,803	143,454,430	143,432,284	123,278,165	699,822,424
N Cardiac Arrest and DNR patients in ED visits	263,961	281,520	265,658	271,015	331,906	1,414,060
% Cardiac Arrest and DNR patients in ED visits	0.18%	0.19%	0.19%	0.19%	0.27%	0.20%
Disposition						
Admitted as inpatient	96,880	108,967	103,819	105,874	142,824	558,364
Mortality in ED	143,189	150,755	139,145	144,113	166,440	743,642
Discharged	10,761	8285	8905	7843	8076	43,870
Transfer to other facility	13,131	13,511	13,789	13,185	14,567	68,183
Mortality						
Did not die	69,502	73,655	74,761	74,821	81,561	374,300
Died in the ED	143,189	150,755	139,145	144,113	166,440	743,642
% Died in the ED	54.25%	53.55%	52.38%	53.18%	50.15%	52.59%
Died in the hospital	51,271	57,110	51,752	52,081	83,905	296,119
% Died in the hospital	52.92%	52.41%	49.85%	49.19%	58.75%	53.03%

Abbreviations: DNR = Do not resuscitate.

**Table 2:** Baseline demographics and characteristics of patients.

Characteristics	Cardiac Arrest and DNR (2016–2020)	
	N	%
N	1,414,062	100.00
Gender	N	%
Female	553,016	39.11
Male	861,046	60.89
Mean Age (Years)	Mean	SD
Female	66.17	16.89
Male	62.67	16.79
Age Groups	N	%
18–29	62,814	4.49
30–49	200,839	14.27
50–69	563,678	39.85
≥70	586,731	41.40
Race (2019–2020)	N	%
Asian or Pacific Islander	15,834	1.13
Black	127,230	8.85
Hispanic	66,544	4.78
Native American	2521	0.17
Other	23,783	1.65
White	367,009	25.42
Disposition from Ed	N	%
Admitted as an inpatient to this hospital	558,364	40.07
Against medical advice	1371	0.10
Died in ED	743,642	52.55
Discharged alive unknown destination	2845	0.20
Home health care	227	0.02
Routine	39,430	2.60
Transfer other	8234	0.56
Transfer to short-term hospital	59,948	3.90
Median Household Income	N	%
≤49,999	499,190	35.30
50,000–64,999	369,425	25.95
65,000–85,999	294,982	20.82
≥86,000	250,465	17.92
Insurance Status	N	%
Medicaid	178,950	12.77
Medicare	777,920	54.79
No charge	4573	0.35
Other	41,071	2.87
Private insurance	267,487	18.86
Self-pay	144,059	10.37
Hospital Region	N	%
Midwest	299,039	20.11
Northeast	253,543	16.91
South Atlantic	630,898	45.20
West	230,581	17.77
Hospital Urban–Rural Designation	N	%
Collapsed category of small metropolitan and micropolitan	12,345	0.74
Large metropolitan areas with at least 1 million residents	727,449	53.34
Metropolitan, collapsed category of large and small metropolitan	11,669	1.07
Micropolitan areas	113,571	7.16
Non-metropolitan, collapsed category of micropolitan and non-urban	26,578	2.11
Not metropolitan or micropolitan (non-urban residual)	64,445	3.92
Small metropolitan areas with less than 1 million residents	458,004	31.66
Hospital Teaching Status	N	%
Metropolitan non-teaching	337,624	26.68
Metropolitan teaching	871,843	60.14
Non-metropolitan hospital	204,595	13.19
Mortality	N	%
Did not die	374,299	26.18
Died in the ED	743,642	52.55
Died in the hospital	296,120	21.26
Comorbidities	N	%
AIDS	7447	0.54
Alcohol	51,877	3.71
Autoimmune	22,382	1.59
Dementia	66,126	4.69
Depression	68,219	4.82
Drug abuse	46,963	3.37
Chronic pulmonary disease	240,537	17.07
Obesity	136,390	9.71
Peripheral vascular disease	68,838	4.86
Hypothyroidism	86,413	6.10
AKI	289,085	20.79
Cardiogenic shock	40,823	2.93
Acute liver failure	58,055	4.16
CAD	302,886	21.47
Smoking	306,179	21.45
HTN	678,861	48.37
Diabetes	387,567	27.66
Cancer	75,065	5.32

AKI: acute kidney injury, HTN: Hypertension, AMI row deleted not required, CAD: Coronary artery disease.

**Table 3:** Predictors of mortality in the emergency department (ED).

Factors	Adjusted Odds Ratio	95% CI Lower Limit	95% CI Upper Limit	*p*-Value
Gender				
Male	Reference			
Female	1	0.97	1.02	0.690
Age Groups				
18–29	Reference			
30–49	1.3	1.22	1.39	<0.001
50–69	1.71	1.6	1.82	<0.001
≥70	2.38	2.23	2.55	<0.001
Race (2019–2020)				
White	Reference			
Asian or Pacific Islander	0.79	0.73	0.85	<0.001
Black	0.96	0.92	0.99	0.008
Hispanic	0.81	0.77	0.84	<0.001
Native American	0.9	0.74	1.1	0.318
Other	0.94	0.88	1	0.047
Median Household Income				
≤49,999	Reference			
50,000–64,999	1.03	1.00	1.06	0.076
65,000–85,999	1.09	1.05	1.13	<0.001
≥86,000	1.12	1.07	1.16	<0.001
Insurance Status				
Medicaid	Reference			
Medicare	1.1	1.05	1.15	<0.001
No charge	1.39	1.14	1.7	0.001
Other	1	0.92	1.08	0.956
Private insurance	0.95	0.91	1	0.031
Self-pay	1.77	1.68	1.86	<0.001
Hospital Region				
Midwest	Reference			
Northeast	0.99	0.95	1.03	0.553
South Atlantic	0.98	0.94	1.01	0.178
West	0.82	0.79	0.86	<0.001
Hospital Urban–Rural Designation				
Large metropolitan areas with at least 1 million residents	Reference			
Collapsed category of small metropolitan and micropolitan	0.98	0.88	1.09	0.663
Metropolitan, collapsed category of large and small metropolitan	1	0.85	1.17	0.988
Micropolitan areas	1.01	0.97	1.06	0.566
Non-metropolitan, collapsed category of micropolitan and non-urban	0.96	0.89	1.05	0.381
Not metropolitan or micropolitan (non-urban residual)	1.19	1.12	1.26	<0.001
Small metropolitan areas with less than 1 million residents	1.03	1	1.06	0.039
Hospital Teaching Status				
Metropolitan teaching	Reference			
Metropolitan non-teaching	1.1	1.07	1.13	<0.001
Non-metropolitan hospital	1.16	1.12	1.21	<0.001
Elixhauser Comorbidities				
≤4	Reference			
>4	1.18	1.01	1.37	0.035

**Table 4:** Common primary diagnoses by ICD-10 and mortality in ED.

Primary Diagnosis	N (%)	Mortality N (%)
Cardiac arrest	761,889 (54%)	659,089 (86.5%)
Respiratory failure	52,223 (3.7%)	15,088 (28.8%)
Gastrointestinal bleed (GIB)	4240 (0.3%)	303 (7.2%)
Syncope	2905 (0.2%)	149 (5.1%)
ST-elevation myocardial infarction (STEMI)	46,248 (3.3%)	1775 (3.8%)
COVID-19	16,952 (1.2%)	625 (3.7%)
Ventricular fibrillation	22,046 (1.6%)	787 (3.6%)
Bradycardia unspecified	2197 (0.2%)	75 (3.4%)
Hyperkalemia	3248 (0.2%)	82 (2.5%)
Illicit drug overdose	10,157 (0.7%)	218 (2.1%)
Pneumonia	7165 (0.5%)	135 (1.9%)
Atrial fibrillation	4041 (0.3%)	66 (1.6%)
Ventricular tachycardia	9661 (1.6%)	148 (1.5%)
Non-ST-elevation myocardial infarction (NSTEMI)	24,373 (1.7%)	244 (1%)
Sepsis	125,303 (8.9%)	990 (0.8%)
Atrioventricular (AV) block complete	6871 (0.5%)	39 (0.5%)

**Table 5:** Detailed breakdown of disposition of patients in the emergency department (ED).

Characteristics	Cardiac Arrest and DNR (2016–2020)				
	Admitted as Inpatient	Mortality in ED	Discharged	Transfer to Other Facility	*p*-Value
N	558,364	743,642	43,872	68,182	
%	39.49	52.59	3.10	4.82	
Gender	%	%	%	%	<0.001
Female	41.98%	50.67%	2.87%	4.48%	
Male	38.84%	53.76%	2.94%	4.45%	
Age in Years	Mean (SD)	Mean (SD)	Mean (SD)	Mean (SD)	
Female	64.94 (16.19)	67.65 (17.24)	64.49 (18.65)	61.89 (16.34)	
Male	62.94 (15.87)	62.79 (17.39)	59.74 (18.13)	60.73 (15.96)	
Age Groups	%	%	%	%	<0.001
18–29	33.90%	56.42%	4.64%	5.05%	
30–49	39.31%	52.01%	3.62%	5.06%	
50–69	42.79%	49.29%	2.76%	5.16%	
≥70	38.39%	55.46%	2.63%	3.52%	
Race (2019–2020)	%	%	%	%	<0.001
Asian or Pacific Islander	48.03%	47.92%	1.93%	2.12%	
Black	44.11%	50.24%	2.55%	3.09%	
Hispanic	49.49%	46.29%	1.62%	2.59%	
Native American	43.29%	46.51%	3.04%	7.16%	
Other	38.72%	55.74%	3.25%	2.29%	
White	39.39%	52.78%	2.44%	5.38%	
Median Household Income	%	%	%	%	<0.001
≤49,999	40.39%	51.75%	3.15%	4.71%	
50K–64,999	39.45%	52.03%	2.86%	5.66%	
65K–85,999	40.66%	52.86%	2.55%	3.93%	
≥86k	39.65%	54.54%	2.94%	2.86%	
Insurance Status	%	%	%	%	<0.001
Medicaid	47.03%	45.12%	3.35%	4.51%	
Medicare	41.97%	51.49%	2.54%	4.01%	
No charge	39.52%	56.00%	1.90%	2.59%	
Other	37.37%	53.92%	3.00%	5.71%	
Private insurance	40.73%	50.56%	3.03%	5.67%	
Self-pay	21.04%	70.48%	4.14%	4.34%	
Hospital Region	%	%	%	%	<0.001
Midwest	37.09%	53.25%	3.54%	6.12%	
Northeast	37.50%	54.88%	3.90%	3.73%	
South Atlantic	40.19%	52.88%	2.65%	4.28%	
West	45.59%	48.72%	1.92%	3.77%	
Hospital Urban–Rural Designation	%	%	%	%	<0.001
Collapsed category of small metropolitan and micropolitan	50.95%	47.34%	1.34%	0.36%	
Large metropolitan areas with at least 1 million residents	42.71%	51.79%	2.91%	2.59%	
Metropolitan, collapsed category of large and small metropolitan	36.04%	57.57%	3.94%	2.45%	
Micropolitan areas	22.58%	58.46%	3.62%	15.34%	
Non-metropolitan, collapsed category of micropolitan and non-urban	36.06%	54.62%	3.73%	5.59%	
Not metropolitan or micropolitan (non-urban residual)	8.64%	66.98%	4.76%	19.62%	
Small metropolitan areas with less than 1 million residents	43.61%	50.53%	2.47%	3.38%	
Hospital Teaching Status	%	%	%	%	<0.001
Metropolitan non-teaching	37.04%	54.65%	2.55%	5.76%	
Metropolitan teaching	45.68%	49.91%	2.84%	1.57%	
Non-metropolitan hospital	20.60%	60.37%	3.98%	15.05%	
Elixhauser Comorbidities	%	%	%	%	<0.001
≤4	38.85%	53.64%	2.96%	4.55%	
>4	92.02%	6.33%	0.67%	0.97%	

**Table 6:** Odds ratios for transfer from the emergency department (ED) versus admission to an inpatient facility.

Factors	Adjusted Odds Ratio	95% CI Lower Limit	95% CI Upper Limit	*p*-Value
Gender				
Male	Reference			
Female	1.08	1.01	1.15	0.029
Age Groups				
18–29	Reference			
30–49	1.12	0.96	1.32	0.150
50–69	1.36	1.17	1.59	<0.001
≥70	1.15	0.97	1.36	0.107
Race (2019–2020)				
White	Reference			
Asian or Pacific Islander	0.47	0.37	0.61	<0.001
Black	0.80	0.73	0.87	<0.001
Hispanic	0.64	0.57	0.73	<0.001
Native American	0.95	0.61	1.47	0.813
Other	0.56	0.45	0.69	<0.001
Median Household Income				
≤49,999	Reference			
50K–64,999	1.30	1.20	1.41	<0.001
65K–85,999	1.17	1.06	1.29	0.002
≥86k	0.93	0.82	1.05	0.229
Insurance Status				
Medicaid	Reference			
Medicare	1.03	0.92	1.16	0.573
No charge	1.23	0.70	2.14	0.471
Other	1.23	1.02	1.49	0.031
Private insurance	1.15	1.03	1.28	0.015
Self-pay	1.56	1.36	1.78	<0.001
Hospital Region				
Midwest	Reference			
Northeast	0.97	0.87	1.09	0.614
South Atlantic	0.51	0.47	0.56	<0.001
West	0.62	0.56	0.69	<0.001
Hospital Urban–Rural Designation				
Large metropolitan areas with at least 1 million residents	Reference			
Collapsed category of small metropolitan and micropolitan	0.08	0.04	0.17	<0.001
Metropolitan, collapsed category of large and small metropolitan	0.42	0.27	0.66	<0.001
Micropolitan areas	2.06	1.88	2.25	<0.001
Non-metropolitan, collapsed category of micropolitan and non-urban	0.63	0.52	0.76	<0.001
Not metropolitan or micropolitan (non-urban residual)	6.76	6.03	7.58	<0.001
Small metropolitan areas with less than 1 million residents	1.19	1.10	1.30	<0.001
Hospital Teaching Status				
Metropolitan teaching	Reference			
Metropolitan non-teaching	4.94	4.57	5.34	<0.001
Non-metropolitan hospital	8.78	8.00	9.64	<0.001
Elixhauser Comorbidities				
≤4	Reference			
>4	2.59	1.77	3.80	<0.001

## Data Availability

All data can be requested from the corresponding author upon reasonable request.

## Supplementary Materials

The following supporting information can be downloaded at: https://www.mdpi.com/article/10.3390/jcm13185585/s1. Table S1. ICD10 Clinical Modification Codes.
