# Supplementary material for: Cardiac Arrest Mortality and Disposition Patterns in United States Emergency Departments
Source: J Clin Med. 2024 Sep 20;13(18):5585. doi: 10.3390/jcm13185585 (PMC11433634; doi:10.3390/jcm13185585)
Supplement: Supplementary file 1 [file jcm-13-05585-s001.zip › jcm-3159887-supplementary.pdf]

Supplementary Table with ICD-10 codes used

Disease/Complications/Comorbidities	ICD-10 Codes
Chronic pulmonary disease	Elixhauser comorbidities index
Diabetes (two types)	Elixhauser comorbidities index
AIDS	Elixhauser comorbidities index
Cancer (five types)	Elixhauser comorbidities index
Dementia	Elixhauser comorbidities index
Autoimmune	Elixhauser comorbidities index
Depression	Elixhauser comorbidities index
Hypothyroidism	Elixhauser comorbidities index
Obesity	Elixhauser comorbidities index
HTN	Elixhauser comorbidities index
Alcohol	Elixhauser comorbidities index
Drug abuse	Elixhauser comorbidities index
Smoking	Elixhauser comorbidities index
Peripheral vascular disease	Elixhauser comorbidities index
AKI	N170,N171,N172,N178,N179,N990
Sudden cardiac arrest	I462,I468,I469
Do not resuscitate	Z66
AMI	I2101,I2102,I2109,I2111,I2119,I2121,
	I2129,I213,I220,I221,I228,I229,I214,I222
Acute liver failure	K7200,K7201,K712
CAD	I2510,I25111,I25118,I25119,I252,I253,I254,
	I2541,I2542,I255,I256,I257,I2570,I25700,
	I25701,I25708,I25709,I2571,I25710,I25711,I25718,
	I25719,I2572,I25720,I25721,I25728,I25729,I2573,I25730,
	I25731,I25738,I25739,I2575,I25750,I25751,I25758,I25759,
	I2576,I25760,I25761,I25768,I25769
Cardiogenic shock	R570
